# Diagnosing sarcopenia in clinical practice: international guidelines vs. population-specific cutoff criteria

**DOI:** 10.3389/fmed.2024.1405438

**Published:** 2024-07-26

**Authors:** Arben Boshnjaku, Ermira Krasniqi

**Affiliations:** ^1^Faculty of Medicine, University “Fehmi Agani” in Gjakova, Gjakovë, Kosovo; ^2^Faculty of Pharmacy, AMECC Rezonanca, Prishtinë, Kosovo

**Keywords:** diagnostic criteria, aging, older people, overdiagnosis, muscle decline

## 1 Introduction

Sarcopenia is a condition characterized by loss of muscle mass and function (1) occurring as a natural part of aging process. Even though initially suggested as a concept to embody the “poverty of flesh” (2), sarcopenia underwent certain transformations throughout its formation, particularly when shifting from muscle mass to muscle strength for the key diagnostic component as suggested by the revised consensus criteria by the European Working Group in Sarcopenia for Older People—EWGSOP2 (1). In its pathway sarcopenia managed to receive an ICD-10-CM code (M62.84) in 2016 (3), thus making a significant step ahead on its establishment and distinction. To date, it's certain and undeniable that sarcopenia presents a topic of ever-growing interest amongst researchers and clinicians, while being relatively unknown amongst the general population. Nonetheless, the continuous increase of expected life expectancy together with aging of worldwide population has inevitably raised the need to include the policy makers and end-users in the matter.

Global prevalence of sarcopenia has been shown to vary widely in between different studies and countries, ranging from 10% using the EWGSOP2 algorithm and diagnostic criteria to 27% using the overall muscle mass definition in a systematic review and meta-analysis involving adults aged ≥ 18 years (4). Another review involving studies with elderly participants while following the most commonly used sarcopenia definitions (EWGSOP, EWGSOP2, Asian Working Group in Sarcopenia—AWGS, International Working Group in Sarcopenia—IWGS and Foundations of National Institutes of Health—FNIH) reported a sarcopenia prevalence ranging between 10 and 16% (5). However, a very comprehensive review including all the available sarcopenia diagnostic approaches that was conducted by Petermann-Rocha et al. (4) showed ranges in outcomes from as low as 0.2% to as high as 86.5% (0.3–91.2% in biological women and 0.4–87.7% in biological men).

It has been a couple of years since Haase et al. (6) addressed the potential implications of sarcopenia diagnosis, particularly arguing about the lack of differentiation of current treatments from the general health recommendations. Elsewhere, Tagliafico et al. (7) even suggested for sarcopenia to be clearly underdiagnosed in clinical practice while arguing for the necessity to involve radiologists in the muscle mass assessment process.

However, despite the fact that the prevalence and diagnosis of sarcopenia remains a topic of increasing interest, the question of how to appropriately diagnose it in clinical practice remains a gray area. Many international working groups have been trying to address this concern, though almost each one ends up bringing novelties to the matter with still no universal consensus guideline. This way, notwithstanding the scientific progress, the greatest issue surrounding sarcopenia remains the lack of its applicability in clinical practices. The reasons remain vague, with the lack of unified diagnostic criteria being above all. Table 1 describes some of the major recommended diagnostic guideline criteria and certain population specific diagnostic cut-off points.

**Table 1:** Recommended diagnostic guideline criteria and population specific cut-off points.

**Working group**	**Muscle mass**	**Muscle function**	
		**Strength**	**Physical performance**
New Mexico Elder Health Survey (8)	•DXA—ASMI •2SD below mean of young reference group	n. a.	n. a.
European Working Group in Sarcopenia for Older People (9)	•DXA—ASMI •♂ < 7.26 kg/m^2^ •♀ < 5.5 kg/m^2^ •BIA—ASMI •♂ < 8.87 kg/m^2^ •♀ < 6.42 kg/m^2^	•Handgrip strength •♂ < 30 kg •♀ < 20 kg	•Gait speed (6 m course) • < 1 m/s •SPPB • ≤ 8 points
Revised European Working Group on Sarcopenia for Older People (EWGSOP2) (1)	•DXA and BIA ASMM •♂ < 20 kg •♀ < 15 kg •ASMI •♂ < 7.0 kg/m^2^ •♀ < 5.5 kg/m^2^	•Handgrip strength •♂ < 27 kg •♀ < 16 kg •Chair stand • < 15 s for 5 rises	•Gait speed • ≤ 0.8 m/s •SPPB • ≤ 8 point score TUG: ≥20 s •400 m walk test •Non-completion or ≥6 min for completion
International working group on sarcopenia (IWGS) (10)	•DXA—appendicular fat lean mass (aLM) to height squared (aLMI/Ht2) •♂ ≤ 7.23 kg/m^2^ •♀ ≤ 5.67 kg/m^2^	n. a.	Gait speed < 1 m/s (4 m course)
The Foundation for the National Institutes of Health (FNIH) (11)	•ALM (BMI adjusted) •♂ < 0.789 •♀ ≤ 0.512 •ALM •♂ < 19.75 kg •♀ < 15.02 kg	•Handgrip strength •♂ < 26 kg •♀ < 16 kg •BMI adjusted grip strength •♂ < 1.0 •♀ < 0.56	n. a.
Asian Working Group on Sarcopenia (AWGS) (12)	•ASMI—DXA •♂ ≤ 7.0 kg/m^2^ •♀ ≤ 5.4 kg/m^2^ •ASMI—BIA •♂ ≤ 7.0 kg/m^2^ •♀ ≤ 5.7 kg/m^2^	•Handgrip strength •♂ < 26 kg •♀ < 18 kg	Gait speed: < 0.8 m/s
Revised Asian Working Group for Sarcopenia (AWGS2)−2019 Consensus Update (13)	•ASMI—DXA •♂ ≤ 7.0 kg/m^2^ •♀ ≤ 5.4 kg/m^2^ •ASMI—BIA •♂ ≤ 7.0 kg/m^2^ •♀ ≤ 5.7 kg/m^2^	•Handgrip strength •♂ < 28 kg •♀ < 18 kg	•Gait speed (6 m course) • < 1.0 m/s •SPPB score • ≤ 9 points •5-time chair stand test ≥12 s
Turkey (14)	•ASMI—BIA •♂ ≤ 8.3 kg/m^2^ •♀ ≤ 5.7 kg/m^2^	•Handgrip strength •♂ < 28 kg •♀ < 14 kg	n. a.
India (15)	•ASMI—DXA •♂ ≤ 6.1 kg/m^2^ •♀ ≤ 4.6 kg/m^2^	•Handgrip strength •♂ < 27.5 kg •♀ < 18 kg	n. a.
Colombia (16)	•ASMI—BIA •♂ ≤ 8.0 kg/m^2^ •♀ ≤ 6.1 kg/m^2^	n. a.	n. a.
Kosovo (17)	•ASMI—BIA •♂ ≤ 5.7 kg/m^2^ •♀ ≤ 4.8 kg/m^2^	•Handgrip strength •♂ < 32.8 kg •♀ < 19.6 kg	•Gait speed •♂ ≤ 1.14 m/s •♀ ≤ 1.03

## 2 Diagnostic approaches of sarcopenia

In principle, there are two common approaches to diagnose sarcopenia: international working group consensus guideline cutoff points vs. population-specific cutoff points. The international working groups cutoff points are based on certain consensus resulting from the agreement between field experts on a specific criterion for the diagnosis of sarcopenia. These specifically-defined criterions are mainly set based on the state-of-the-art research evidence from patterns deriving from one or more populations with similar characteristics, or from big and comprehensive studies were performed in the similar populations. However, the population-specific ones are tailored-based diagnostic criteria deriving from the population specifics, generated on the distribution of muscle mass, strength and physical performance amongst the specific population. They are calculated by determining the standard deviation from the respective mean of a given parameter within a younger population group, and then subtracting that standard deviation (usually from 2 to 2.5 times) from a respective mean population, thus defining a cut point that represents a certain number of standard deviations below the mean (8).

As observed in Table 2, both approaches have their own advantages and disadvantages of (and when) being followed. Unfortunately, certain limitations that come in the expense of an extra benefit are undeniably worrisome, like the case of standardization in between populations that the international guidelines endure in expense to the demography accuracy. A similar case is as well-observed with clinical relevancy and research accuracy that the population-specific cutoff points provide, though in the expense of practicality and consistency.

**Table 2:** Advantages and disadvantages of international consensus vs. population-specific diagnostic criteria for sarcopenia.

	**International guidelines**	**Population-specific**
Advantages	•Standardization •Consensus-derived •Consistency •Comparability •Practicality •Evidence-based •Facilitates clinical decision-making	•Demographically accurate •Scientifically accurate •Clinically relevant •Personalized assessments •Empowers patients through personal approach •Facilitates tailored-based public health interventions
Disadvantages	•Limited population specificity •Limited flexibility •Limited cultural and socio-economic influence consideration •Erroneous representation within diverse populations •Under/overdiagnosis	•No standardized guidelines •Need for perpetual updating •Complexity •Low generalizability •Interprofessional (healthcare) misunderstandings •Under/overdiagnosis

Notwithstanding the applicability of both approaches, the internationally-suggested criterions are generally considered as more reliable and valid when dealing with prevalence/epidemiological studies, or studies within big populations with similar characteristics. To some degree this is understandable based on the standardization they provide, resulting in a more practical applicability (while not having to keep developing population-specific diagnostic criteria), as well as a generalizability (encompassing different populations) and consistency (by following the same pathway). On the contrary to that, population-specific criteria might raise concerns on their reliability, mainly due to their high reliance on the distribution of muscle mass and strength within a specific population, as something that can vary widely across different geographic regions (latitude) and demography (ethnicity, biological gender, age). Though if population specific diagnostic criteria are derived from a well-planed and high-end customization study conducted within a particular population, the certainty of diagnostic pathway could be very high. In certain cases, even the international guidelines do suggest to use regional normative populations (when available), particularly when dealing with variables prone to stature variations like strength and gait speed (1). Unfortunately, this is often not the case in scientific practice, particularly amongst developing countries where age-related diseases like sarcopenia are rather neglected in comparison to others like emergency medicine and acute medical interventions that often take the spotlight (18). Such situations seriously limit researchers and/or practitioners' available possibilities to diagnose and perform in their practice. This way the comparability between different populations (in particular) becomes obsolete due to the specific circumstances around them. Nonetheless, if there is an outcome deriving from the current state-of-the-art, it seems that both approaches do provide the ground for either under or over diagnosis of sarcopenia and its conceptual stages.

## 3 Which approach should we follow?

Both, mostly depending on the context. It is a fact that we cannot develop population-specific diagnostic criteria for every disease out there! Though in clinical practice, the most important factor is to find cases with potential to develop disease. If the population-specific diagnostic cut-off points result from a comprehensive epidemiological study that considers other inter-related covariates like the individual health, environment and lifestyle behaviors, they most definitely provide the more accurate diagnostic pathway. It is particularly true for the un-/under—explored populations (developing countries) or for the small populations (e.g., small countries). Therefore, population specific diagnostic criteria should be the frontline to be used in clinical practices, for as long as they would have a strong “backbone” criterion-derived study from which they would be based (17, 19). Then, sarcopenia diagnosis should be followed by other means of diagnostic approach including qualitative muscle assessments (echogenicity analysis) through diagnostic ultrasound to provide insights within the pathophysiology of sarcopenia (20), as a reliable and valid diagnostic method for quantitative assessment of age-related changes in appendicular muscle mass (21). And this is the momentum which we would need to catch since the skeletal muscle provides the qualitative context besides the quantitative one. It is particularly important to visualize and better understand the qualitative muscle changes that accompany the sarcopenia diagnosis (and/or its conceptual stages).

The revised EWGSOP2 consensus definition and diagnostic algorithm (1) has been one of the more frequent and novel diagnostic pathways followed in many recent studies (though predominantly coming from European populations since being developed for these populations). The standard diagnostic flowchart suggests to start with the sarcopenia screening questionnaire (SARC-F) for potential case findings, and proceed with muscle strength assessment by either isometric grip strength (assessed by dynamometry) or lower body strength (assessed by chair stand test). When either of strength parameters is below the threshold the term “sarcopenia probable” is given, which requires to undergo the sarcopenia confirmation process that involves the assessment of appendicular skeletal muscle mass (or index) through one of the recommended techniques (either dual x-ray energy absorptiometry—DXA, or bio electrical impedance analysis—BIA). If the outcomes are below the set thresholds in this phase as well, estimating the severity of sarcopenia through physical performance assessment (gait speed at normal walking pace) remains the final step. In this context, the international consensus guidelines are definitely important for the populations they encompass or that were taken into consideration when being developed, and they should be used in research, for prevalence studies and in addition to the other assessment approach. Their potential population specificity limitations could be overcome with the qualitative approach as well. Though, sarcopenia in clinical practice shouldn't be just about filling or not some certain criteria, but rather as a comprehensive geriatric condition that needs to be detected and tackled accordingly. However, until a unified worldwide diagnostic pathway to follow is brought up, clinicians are most definitely going to hesitate to move forward toward mass diagnosing. Until then, diagnosis of sarcopenia should be made carefully following both approaches (international and population-specific criteria), always evaluated in conjunction with a comprehensive medical evaluation, including a physical exam, medical history, and laboratory tests, as well as followed by muscle echogenicity analysis to estimate muscle quality. This should be important particularly in developing countries where numbers of sarcopenia cases are expected to raise with time going by, whereas normative data remain inconclusive (18). In this context, the inclusion of certain experts from such countries (notwithstanding the size of the representative population) within international working groups might offer other (different) perspectives on the matter. This might allow clinician to make decisions based on gathered evidences from different facts, thus giving some time for the more appropriate sarcopenia-diagnostic approach to settle. After all, if the sarcopenia diagnosis does not start to be applied in clinical practice, it risks becoming an obsolete. It might even take the path of overdiagnosis (6) and regress from the brave steps taken to date.

## Author contributions

AB: Conceptualization, Methodology, Resources, Visualization, Writing – original draft. EK: Conceptualization, Project administration, Writing – review & editing.
